# Ectonucleotidase-Mediated Suppression of Lupus Autoimmunity and Vascular Dysfunction

**DOI:** 10.3389/fimmu.2018.01322

**Published:** 2018-06-11

**Authors:** Jason S. Knight, Levi F. Mazza, Srilakshmi Yalavarthi, Gautam Sule, Ramadan A. Ali, Jeffrey B. Hodgin, Yogendra Kanthi, David J. Pinsky

**Affiliations:** ^1^Division of Rheumatology, Department of Internal Medicine, University of Michigan, Ann Arbor, MI, United States; ^2^Department of Pathology, University of Michigan, Ann Arbor, MI, United States; ^3^Division of Cardiology, Ann Arbor Veterans Administration Healthcare System, Ann Arbor, MI, United States; ^4^Division of Cardiovascular Medicine, Department of Internal Medicine, University of Michigan, Ann Arbor, MI, United States; ^5^Department of Molecular and Integrative Physiology, University of Michigan, Ann Arbor, MI, United States

**Keywords:** systemic lupus erythematosus, ectonucleotidases, CD73, CD39, T_H_17 cells, endothelial dysfunction, neutrophil extracellular traps

## Abstract

**Objectives:**

CD39 and CD73 are surface enzymes that jut into the extracellular space where they mediate the step-wise phosphohydrolysis of the autocrine and paracrine danger signals ATP and ADP into anti-inflammatory adenosine. Given the role of vascular and immune cells’ “purinergic halo” in maintaining homeostasis, we hypothesized that the ectonucleotidases CD39 and CD73 might play a protective role in lupus.

**Methods:**

Lupus was modeled by intraperitoneal administration of pristane to three groups of mice: wild-type (WT), CD39^−/−^, and CD73^−/−^. After 36 weeks, autoantibodies, endothelial function, kidney disease, splenocyte activation/polarization, and neutrophil activation were characterized.

**Results:**

As compared with WT mice, CD39^−/−^ mice developed exaggerated splenomegaly in response to pristane, while both groups of ectonucleotidase-deficient mice demonstrated heightened anti-ribonucleoprotein production. The administration of pristane to WT mice triggered only subtle dysfunction of the arterial endothelium; however, both CD39^−/−^ and CD73^−/−^ mice demonstrated striking endothelial dysfunction following induction of lupus, which could be reversed by superoxide dismutase. Activated B cells and plasma cells were expanded in CD73^−/−^ mice, while deficiency of either ectonucleotidase led to expansion of T_H_17 cells. CD39^−/−^ and CD73^−/−^ mice demonstrated exaggerated neutrophil extracellular trap release, while CD73^−/−^ mice additionally had higher levels of plasma cell-free DNA.

**Conclusion:**

These data are the first to link ectonucleotidases with lupus autoimmunity and vascular disease. New therapeutic strategies may harness purinergic nucleotide dissipation or signaling to limit the damage inflicted upon organs and blood vessels by lupus.

## Introduction

Systemic lupus erythematosus (commonly referred to as “lupus”) is the prototypical systemic autoimmune disease. In the United States, the prevalence of lupus approaches 1 in 500, with a disproportionate impact on women of childbearing age and minorities. The immunopathology of lupus is complex, with derangements present in both lymphocyte- and myeloid-lineage cells. Beyond the well-recognized damage inflicted by lupus upon organs such as kidneys, skin, and joints, lupus cardiovascular disease has emerged as a leading cause of morbidity and mortality. Indeed, young women with lupus carry a 50-fold increased risk of cardiovascular events when compared with their unaffected peers (1).

Although the proximal trigger for lupus is unknown, there is some evidence that environmental factors are contributory. In mice, the intraperitoneal administration of pristane (a naturally occurring hydrocarbon) recruits inflammatory macrophages into the peritoneal cavity, where they robustly produce type I interferons (2, 3). Mediated at least in part by these interferons, female mice take on features of lupus over the ensuing 6–9 months, including anti-ribonucleoprotein (anti-RNP) antibody production, splenic immune cell derangements, and immune complex glomerulonephritis (2, 3). Mechanistically, the pristane model of lupus depends upon both the type I interferon receptor and toll-like receptor 7 for autoantibody formation and other aspects of the lupus phenotype (4, 5). In recent years, the pristane model has been used to assess wide-ranging concepts in lupus pathogenesis including the roles of leptin (6), selectin-mediated leukocyte adhesion (7), and the inflammasome (8).

Leukocytes and endothelial cells are regulated by a dynamic halo of ATP, ADP, AMP, and adenosine. Purine nucleotides are liberated in large quantities from dying cells at sites of hypoxic, ischemic, or inflammatory stress (9). ATP and ADP then engage cell-surface receptors to launch proinflammatory and prothrombotic cascades (9). By contrast, adenosine (the extracellular concentration of which can rise by orders of magnitude during acute inflammation) has potent antithrombotic, anti-inflammatory, and immunosuppressive properties mediated by surface G protein-coupled receptors (10).

**Figure 1:**
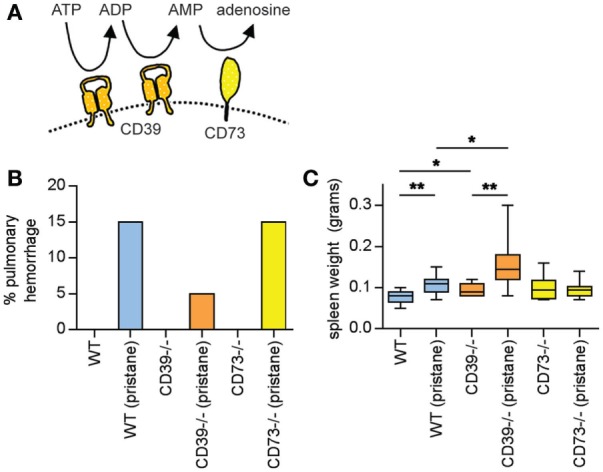
Pulmonary hemorrhage and splenomegaly in pristane-treated mice. **(A)** Schematic of CD39 and CD73, which together mediate the step-wise phosphohydrolysis of extracellular ATP into adenosine. **(B)** Some mice developed clinically overt pulmonary hemorrhage within the first month after pristane administration; these mice required euthanasia. *N* = 10 saline-treated mice and 20 pristane-treated mice per genotype; no comparisons were statistically significant. **(C)** Mice were administered either saline or pristane, as indicated. 36 weeks later, spleen size was measured. *N* = 10 per control group and 17–19 per pristane group; *p < 0.05 and **p < 0.01. Box-and-whisker plots denote minimum, 25th percentile, median, 75th percentile, and maximum.

To regulate the local concentrations of purine nucleotides and adenosine, the ectonucleotidases CD39 and CD73 extend into the extracellular space from the surfaces of leukocytes and endothelial cells (Figure 1A). CD39 is a membrane-spanning enzyme with an ectodomain that cleaves the terminal phosphate group from ATP to form ADP, and then from ADP to form AMP (11). From there, CD73 (a GPI-anchored protein) clips the final phosphate group from AMP to generate adenosine (11). The endothelium is a key site of ectonucleotidase expression, with well-recognized upregulation in response to stressors such as hypoxia, thereby limiting leukocyte activity and efflux (12–14). Leukocytes (including lymphocytes and neutrophils) also express ectonucleotidases, not only to autoregulate activation, adhesion, and transit but also to manipulate neighboring cells (15, 16). Indeed, specialized immune cells such as regulatory T cells (Tregs) and myeloid-derived suppressor cells mediate their effects in part through local ectonucleotidase-generated adenosine (15, 16). To the best of our knowledge, the only connection between ectonucleotidases and lupus reported to date is the observation that some lupus patients lack adequate T-cell expression of CD39 and CD73, hinting at a defect in regulatory T-cell function (17, 18). The studies described here seek to provide new insight into how a pathway that functions as an endogenous guardian against inflammation may be exploited to counteract lupus.

## Materials and Methods

### Animal Housing and Treatments

Mice were housed in a specific pathogen-free barrier facility, and fed standard chow. Female C57BL/6 mice were purchased from The Jackson Laboratory. CD39^−/−^ mice have been described by our group previously (19). CD73^−/−^ mice in the C57BL/6 background were originally obtained from Dr. Linda Thompson and have been used by our group previously (20). Pristane was purchased from Sigma. At 8–10 weeks of age, female mice were administered a single intraperitoneal dose of 500 µl pristane or 500 µl normal saline. Unless otherwise indicated, studies were performed on mice euthanized at 36 weeks. This study was carried out in accordance with the recommendations of the National Research Council, Guide for the Care and Use of Laboratory Animals. The protocol was approved by the Institutional Animal Care and Use Committee.

### Complete Blood Counts

Peripheral leukocyte and platelet counts were determined with an automated Hemavet 950 counter (Drew Scientific).

### ELISAs

Kits for mouse anti-nRNP IgG (5415) and mouse total IgG (6320) were purchased from Alpha Diagnostic International and performed according to the manufacturer’s instructions.

### Kidney Scoring

At the time of euthanasia, kidneys were gently perfused with heparinized saline. A portion of the cortex was frozen in Tissue-Tek OCT (Sakura Finetek) for immunofluorescence staining. Staining for kidney IgG and C3 was performed on frozen sections as described (21, 22). Another portion of the cortex was fixed in formalin and embedded in paraffin. Formalin-fixed sections were stained by periodic acid-Schiff and then scored in a blinded manner as previously described (21, 22). In brief, a semiquantitative scoring system: 0, no involvement; 0.5, minimal involvement of <10%; 1, mild involvement (10–30% section); 2, moderate involvement (31–60% of section); and 3, severe involvement. This system was used to assess 13 different parameters of activity and chronicity (mesangial hypercellularity, mesangial deposits, mesangial sclerosis, endocapillary cellular infiltrate, subepithelial and subendothelial deposits, capillary thrombi, capillary sclerosis, cellular or organized crescents, synechiae, tubular atrophy, and interstitial fibrosis). For glomerular indices, 30 glomeruli were examined per mouse, and a cumulative score was determined for each parameter.

### Flow Cytometry

A single-cell suspension of splenocytes was analyzed with the following anti-mouse antibodies (all from BioLegend): CD138, B220, CD80, CD19, CD44, and CD62L. Mouse Th1/Th17 Phenotyping Kit was from BD Pharmingen and performed according to the manufacturer’s instructions. Staining was typically for 30 min at 4°C. After washing, cells were fixed in 2% paraformaldehyde before analysis with a CyAn ADP Analyzer (Beckman Coulter). Further data analysis was done using FlowJo analysis software.

### Endothelial Function

Studies were performed as previously reported by our group (21, 22). After euthanasia with pentobarbital, thoracic aortas were excised, cleaned, and cut into 2-mm length rings. The endothelium was left intact and rings were mounted in a myograph system (Danish Myo Technology A/S). Rings were bathed with warmed and aerated (95% O_2_/5% CO_2_) physiological salt solution. Aortic rings were set at 7 mN passive tension and equilibrated for 1 h. Cumulative doses of phenylephrine (10^−9^ to 10^−6^ M) were then added to the bath to establish a concentration-response curve. After washing, a phenylephrine concentration corresponding to 80% of the maximum was added, and contraction was allowed to reach a stable plateau. To examine endothelium-dependent relaxation, acetylcholine (10^−9^ to 10^−6^ M) was added cumulatively to the bath. Finally, a normal vascular smooth muscle response was confirmed by washing out phenylephrine and acetylcholine, and then repeating the experiment with phenylephrine contraction followed by cumulative addition of sodium nitroprusside (10^−9^ to 10^−5^ mol/L). In some experiments, one aortic ring from each mouse was treated as usual, while a second ring was incubated with superoxide dismutase (SOD) 1.2 kU/ml during the equilibration phase of the experiment.

### Blood Pressure

Non-invasive blood pressure was measured by tail cuff as described (23). Briefly, using the IITC Life Science blood pressure measurement system, conscious and restrained mice were acclimated for 3 days in a temperature controlled environment (model 306 warming chamber). The tail vein was occluded with an integrated sensor-cuff (model I-B60-1/4) and return of pulsation (RTP) detected by the RTP-computerized blood pressure monitor (model 6M 229 6 channel mouse system). Repeated measures were averaged for determination and report of systolic blood pressure and heart rate.

### Quantitative PCR

At the time of tissue harvest, aortas were snap frozen in liquid nitrogen and stored at −80°C. Later, the aortas were mechanically homogenized in TriPure Isolation Reagent (Roche). RNA was prepared by the Direct-zol RNA MiniPrep kit (Zymo Research) according to the manufacturer’s instructions. RNA integrity number was >7 for all included samples. cDNA was synthesized using MMLV RT (Invitrogen) and 100 ng of RNA using a MyCycler thermocyler (Bio-Rad). Quantitative PCR was with SYBR Green PCR Master Mix (Applied Biosystems) according to the manufacturer’s instructions, and carried out using an ABI PRISM 7900HT (Applied Biosystems). Primers for the housekeeping gene beta-actin were purchased from Qiagen (QuantiTect Primer Assays, which have proprietary primer sequences). Primer sequences for endothelial nitric oxide synthase (eNOS) were 5′-GACCCTCACCGCTACAACAT-3′ and 5′-TTTGGCCAGCTGGTAACTGT-3′; primer sequences for inducible NOS (iNOS) were 5′-TGGTGGTGACAAGCACATTT-3′ and 5′-GCCAAACACAGCATACCTGAA-3′. Ct values were normalized to the housekeeping gene to determine ΔCt. ΔΔCt values were then determined by comparing each ΔCt to the average ΔCt for the wild-type (WT) control group. Data were presented as relative fold change by the formula 2^ΔΔCt^.

### Measurement of Cell-Free DNA

Cell-free DNA was quantified in plasma using the Quant-iT PicoGreen dsDNA Assay Kit (Invitrogen) according to the manufacturer’s instructions.

### Neutrophil Purification and NETosis Assay

Bone marrow neutrophils were isolated as previously described (21, 22). Briefly, total bone marrow cells were spun on a discontinuous Percoll gradient (52–69–78%) at 1,500 × *g* for 30 min. Cells from the 69–78% interface were collected. These cells were >95% Ly-6G-positive by flow cytometry and had typical nuclear morphology by microscopy. To assess *in vitro* NETosis, a protocol similar to what we have described previously was followed (21, 22). Culture was for 4 h at 37°C in RPMI media supplemented with 2% bovine serum albumin and 10 mM HEPES buffer. Stimulation with phorbol-12-myristate-13-acetate (100 nM, Sigma) was also for 4 h. For immunofluorescence, cells were fixed with 4% paraformaldehyde. DNA was stained with Hoechst 33342 (Invitrogen), while protein staining was with rabbit polyclonal antibody to citrullinated histone H3 (Abcam), followed by FITC-conjugated anti-rabbit IgG (SouthernBiotech). Images were collected with an Olympus IX70 microscope and a CoolSNAP HQ2 monochrome camera (Photometrics) with Metamorph Premier software (Molecular Devices). Neutrophil extracellular traps (NETs) (decondensed areas of extracellular DNA co-staining with one of the aforementioned protein markers) were quantified by two blinded observers, and digitally recorded to prevent multiple counts; the percentage of NETs was calculated after counting 10 400× fields per sample.

### Statistical Analysis

Data analysis was with GraphPad Prism software version 6. For continuous variables, normally distributed data were analyzed by unpaired two-tailed *t* testing, while skewed data were assessed by Mann–Whitney test. For dichotomous variables, analysis was by Chi square. For endothelial function experiments, curve fit was by the least squares method, and comparisons were by two-way ANOVA. Statistical significance was defined as *p* < 0.05.

## Results

The one-time intraperitoneal administration of the natural hydrocarbon pristane promotes features of lupus, which emerge over 6–9 months (2, 3). Here, pristane was administered to three groups of mice at 8–10 weeks of age, all in the C57BL/6 background: WT, CD39^−/−^, and CD73^−/−^. Twenty mice were treated with pristane and 10 with saline for each genotype.

A feature of pristane administration to C57BL/6 mice is the induction of diffuse alveolar hemorrhage, which becomes clinically apparent in 15–20% of mice approximately 4 weeks after treatment (24, 25). Mechanistically, roles have been suggested for both B cells (24) and macrophages (25) in the pulmonary-hemorrhage phenotype. Here, three WT mice (15%), one CD39^−/−^ mouse (5%), and three CD73^−/−^ mice (15%) developed clinically overt pulmonary hemorrhage in response to pristane (and required euthanasia), all within 4 weeks of pristane administration (Figure 1B). No saline-treated mouse developed a similar phenotype (Figure 1B). All remaining mice survived to 36 weeks, at which time they were euthanized.

### CD39 Deficiency Potentiates Splenomegaly in Response to Pristane

At 36 weeks, pristane promoted splenomegaly in WT mice, which was further potentiated by CD39 deficiency (Figure 1C).

### Ectonucleotidase Deficiency Potentiates Autoimmunity in Pristane-Treated Mice

**Figure 2:**
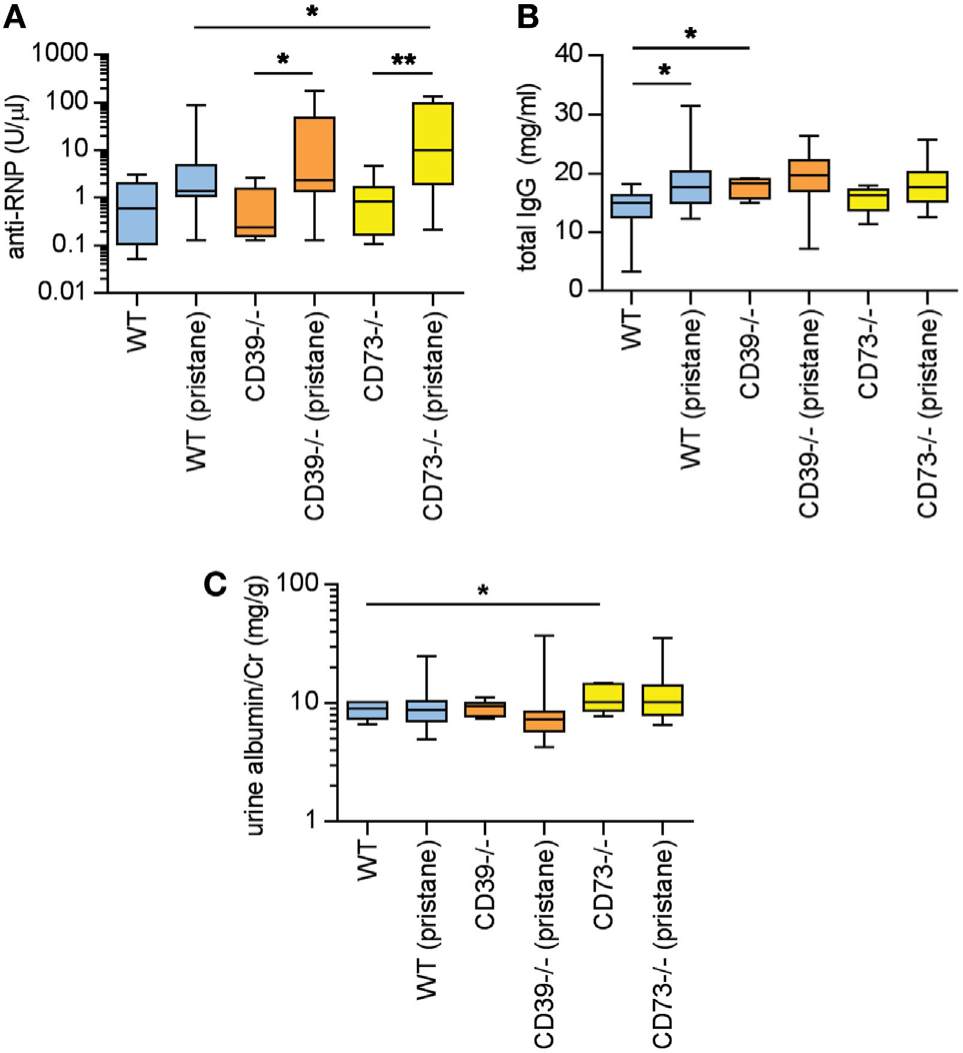
Ectonucleotidase deficiency potentiates autoimmunity in pristane-treated mice. Mice were administered either saline or pristane, as indicated. 36 weeks later, various endpoints were tested. **(A)** Anti-ribonucleoprotein (Anti-RNP) IgG was measured in serum. **(B)** Total IgG was measured in serum. **(C)** Spot albumin/Cr (albumin/creatinine) ratios in urine. *N* = 10 per control group and 17–19 per pristane group. *p < 0.05 and **p < 0.01.

Pristane administration induces the production of autoantibodies, especially to ribonucleoproteins (RNPs) (2, 3). Here, serum anti-RNP antibody levels were measured 36 weeks after pristane administration. While WT mice demonstrated a strong trend toward induction of anti-RNP antibodies with pristane administration (*p* = 0.054), this induction was further potentiated by ectonucleotidase deficiency (Figure 2A). Anti-RNP antibodies were not detected at a significant level in any of the saline-treated groups (Figure 2A). We also measured serum total IgG. In WT mice, pristane triggered higher levels of IgG when compared with saline-treated controls (Figure 2B). A trend for increased IgG was also observed in the ectonucleotidase-deficient mice, although this did not reach statistical significance (which may have been at least partially attributable to a higher “baseline” in the ectonucleotidase-deficient controls) (Figure 2B). In summary, these data indicate that ectonucleotidase deficiency potentiates autoantibody formation, but not total IgG levels, in response to pristane.

### C57BL/6 Mice Do Not Develop Significant Proteinuria in Response to Pristane

In previous studies of pristane administration to C57BL/6 mice, the kidneys have demonstrated a relatively mild phenotype of increased mesangial cellularity, which is compatible with World Health Organization Class II lupus nephritis in patients (2, 26). Immune complex and complement deposition have also been appreciated in pristane-treated C57BL/6 mice, albeit in the setting of minimal proteinuria (2, 26). Here, the albuminuria detected at 36 weeks was at a level that would be considered microalbuminuria (i.e., albumin/creatinine <300 mg/g) (Figure 2C); the only statistically significant difference between groups was that saline-treated CD73^−/−^ mice demonstrated higher levels of microalbuminuria than saline-treated WT mice. Beyond albuminuria, kidney glomeruli were scored for IgG and C3 deposition at 36 weeks. Pristane administration did enhance both IgG and C3 deposition when compared with saline-treated controls (Figures S1A–C in Supplementary Material); however, ectonucleotidase deficiency did not potentiate either IgG or C3 deposition. Kidney histopathology was also assessed across a variety of inflammatory parameters (see Materials and Methods). As compared with saline-treated controls, WT mice demonstrated an increase in mesangial hypercellularity upon pristane administration (Figures S1D,E in Supplementary Material); there were no other statistically significant differences between the groups. In summary, there is evidence that pristane administration induces modest glomerular immune complex deposition and mesangial hypercellularity, albeit without significant proteinuria, and without potentiation by ectonucleotidase deficiency.

### Ectonucleotidase Deficiency Expands B- and T-Cell Populations

**Figure 3:**
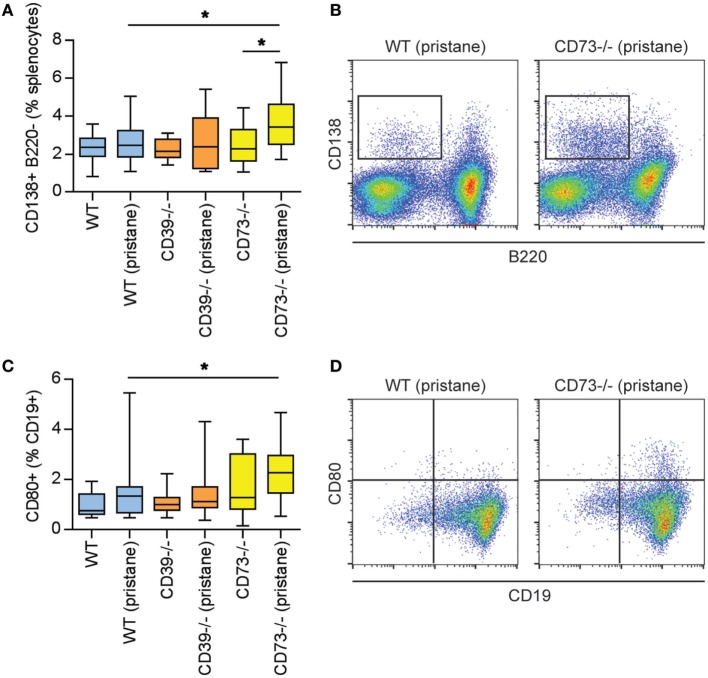
Modulation of plasma cells and B cells by ectonucleotidase deficiency in pristane-treated mice. Mice were administered either saline or pristane, as indicated. 36 weeks later, splenocytes were analyzed by flow cytometry. **(A)** CD138^+^ B220^−^ plasma cells, presented as the percentage of total splenocytes. **(B)** Representative data as presented in panel **(A)**. **(C)** CD80^+^ activated B cells, presented as the percentage of CD19^+^ B cells. **(D)** Representative data as presented in panel **(C)**. *N* = 10 per control group and 17–19 per pristane group; *p < 0.05.

**Figure 4:**
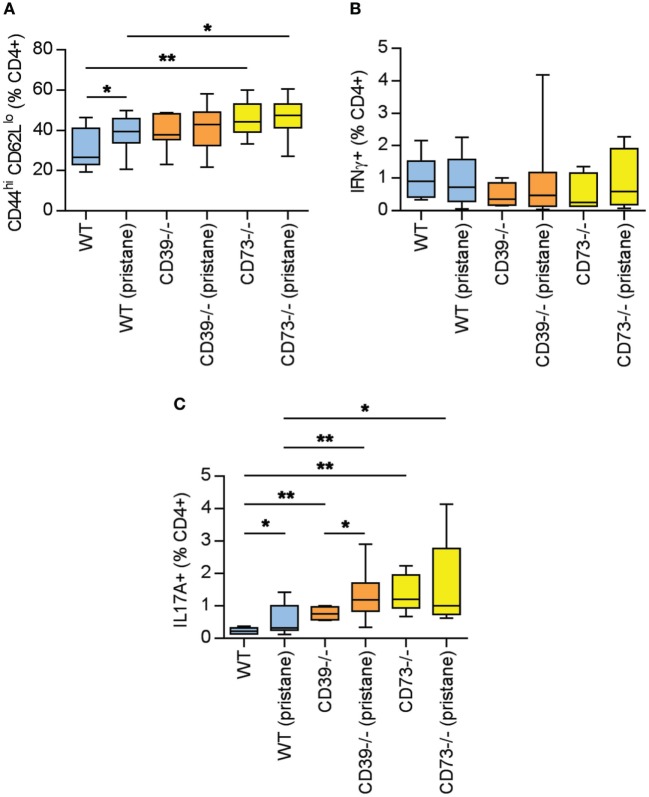
Potentiation of T cell activation and T_H_17 polarization by ectonucleotidase deficiency. Mice were administered either saline or pristane, as indicated. 36 weeks later, splenocytes were analyzed by flow cytometry. **(A)** CD44^hi^ CD62L^lo^ effector/memory T cells, presented as the percentage of CD4^+^ T cells. **(B)** Percentage of CD4^+^ T cells expressing interferon gamma. **(C)** Percentage of CD4^+^ T cells expressing interleukin 17A. For panel **(A)**, *n* = 10 per control group and 17–19 per pristane group. For panels **(B,C)**, *n* = 5 per control group and 8–10 per pristane group; *p < 0.05 and **p < 0.01.

As above, there is evidence of exacerbated autoimmunity (splenomegaly, increased anti-RNP antibodies) when ectonucleotidase-deficient mice are treated with pristane. To understand this mechanistically, we first characterized splenic B cell populations. As compared with WT mice, CD73^−/−^ mice demonstrated expansion of plasma cells (Figures 3A,B) and CD80^+^ activated B cells (Figures 3C,D); a similar expansion was not seen in CD39^−/−^ mice. Moreover, as compared with pristane-treated WT mice, there was an increase in splenic follicular and marginal zone B cells in pristane-treated CD73^−/−^ mice (Figure S2 in Supplementary Material). We also characterized splenic T cells. As compared with pristane-treated WT mice, there was an expansion of effector/memory CD4^+^ T cells (CD44^hi^ CD62L^lo^) in pristane-treated CD73^−/−^ mice (Figure 4A); interestingly, this increase was also noted in the saline-treated CD73^−/−^ controls. While there was no difference in IFNγ-producing T_H_1 cells (Figure 4B), IL-17A-producing T_H_17 cells were significantly increased in both CD39^−/−^ and CD73^−/−^ mice (Figure 4C). The T_H_17 expansion was robust—present at baseline (i.e., the saline groups) and then further potentiated by pristane (Figure 4C). In summary, these data demonstrate that ectonucleotidase deficiency promotes expansion of T_H_17 cells, while plasma cells and activated B cells are expanded only in the CD73-deficient mice.

### Ectonucleotidases Protect Against Endothelial Dysfunction in Pristane-Treated Mice

**Figure 5:**
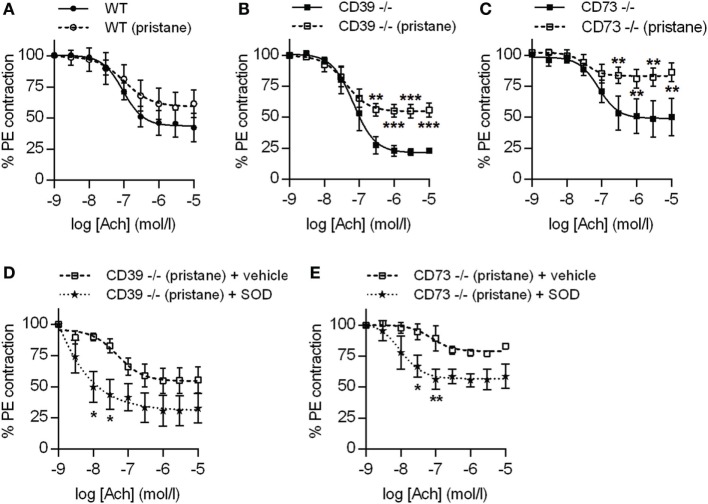
Ectonucleotidase deficiency potentiates endothelial dysfunction in pristane-treated mice. Mice were administered either saline or pristane, as indicated. 36 weeks later, “aortic rings” were harvested for *ex vivo* determination of endothelial function by measuring the contractile force remaining in pre-contracted (by phenylephrine/PE) aortic rings in response to progressively increasing concentrations of acetylcholine. A “deeper” curve indicates a healthier endothelium, while a “flatter” curve denotes endothelial dysfunction. **(A–C)** *N* = 5 control mice and 7 pristane mice per graph. **(D,E)** Two aortic rings were isolated from each mouse (*n* = 5), and one was treated with superoxide dismutase (SOD). *p < 0.05, **p < 0.01, and ***p < 0.001.

Dysfunction of the arterial endothelium (as defined by impaired flow-mediated dilation) is a harbinger of atherosclerosis in lupus patients (27). Endothelial dysfunction is driven by oxidative stress, under which nitric oxide synthase becomes “uncoupled” and produces vasoconstrictive superoxide anion rather than vasodilatory nitric oxide (27). In both CD39^−/−^ and CD73^−/−^ mice (but not WT mice), pristane administration induced significant dysfunction of the arterial endothelium, when compared with their respective control groups (Figures 5A–C). Interestingly, CD39^−/−^ mice demonstrated a trend toward more robust baseline aorta relaxation, when compared with the other two genotypes (Figures 5A–C). The endothelial dysfunction of CD39^−/−^ and CD73^−/−^ mice was not explained by alterations in either blood pressure or heart rate (Figure S3 in Supplementary Material) and could be mitigated by *ex vivo* administration of SOD to aortic rings (to neutralize reactive oxygen species) (Figures 5D,E). We also assessed transcription of the genes for both eNOS and iNOS in aortas and found that eNOS transcription was upregulated in CD39^−/−^ and CD73^−/−^ mice when compared with WT mice, but was not further modified by pristane administration (Figure S4A in Supplementary Material). Transcription of iNOS was not significantly regulated by any of the conditions (Figure S4B in Supplementary Material). In summary, these data demonstrate that ectonucleotidases protect against dysfunction of the arterial endothelium in response to pristane, and that pristane-induced dysfunction can be rescued by administration of SOD.

### Ectonucleotidase Deficiency Potentiates Neutrophil Activation

**Figure 6:**
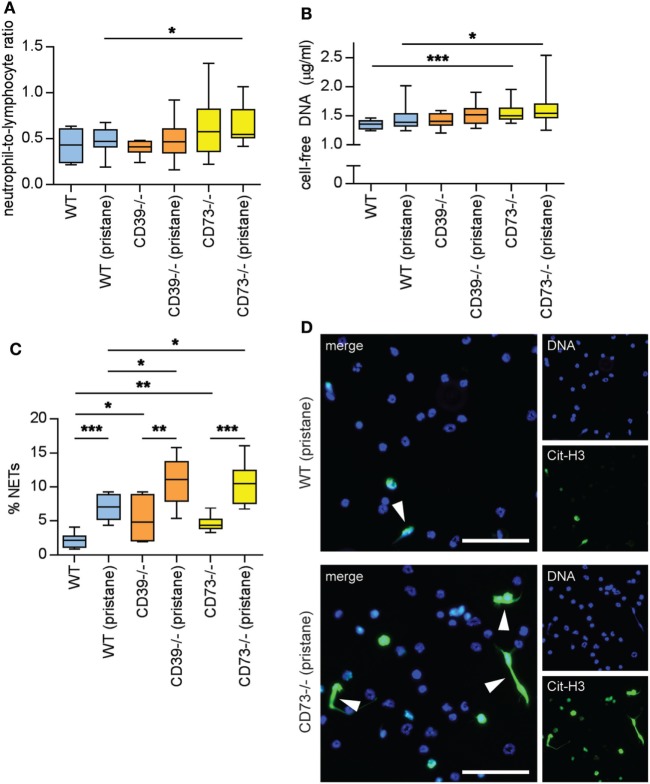
Ectonucleotidase deficiency potentiates neutrophil activation. Mice were administered either saline or pristane, as indicated. 36 weeks later, various endpoints were assessed. **(A)** Neutrophil-to-lymphocyte ratio in peripheral blood. **(B)** Cell-free DNA in mouse serum. **(C)** Mature neutrophils were purified from mouse bone marrow and cultured for 4 h on polylysine-coated glass coverslips. Spontaneous neutrophil extracellular trap (NET) release was assessed by immunofluorescence microscopy. **(D)** Representative photomicrographs from the data presented in panel C. DNA is stained blue and citrullinated histone H3 (Cit-H3) green. NETs are identified as extracellular areas of blue and green overlap (arrowheads). Scale bar = 50 µm. For panels **(A,B)**, *n* = 10 per control group and 17–19 per pristane group. For panel **(C)**, *n* = 6–9 per group; *p < 0.05, **p < 0.01, and ***p < 0.001.

Neutrophils are being increasingly recognized as pathogenic agents in lupus (28, 29). For example, a “neutrophil signature” in blood heralds the onset of lupus nephritis in lupus patients (30, 31). NETs released by lupus neutrophils are at least one driver of type I interferon production (which counteracts endothelial homeostasis) (32, 33), while hyperactive neutrophils are likely an important stressor of the lupus endothelium (21, 22, 34). As compared with WT mice, CD73^−/−^ mice demonstrated an increased neutrophil-to-lymphocyte ratio, especially in response to pristane (Figure 6A); the increased ratio was related to an increase in absolute neutrophil count, more so than a decrease in the lymphocyte count (Figure S5 in Supplementary Material). CD73^−/−^ mice also demonstrated elevated levels of cell-free DNA (a surrogate for NETs) in serum (Figure 6B). *Ex vivo*, NET release was accelerated by deficiency of either CD39 or CD73, both at baseline (i.e., in the saline groups) and in response to pristane (Figures 6C,D). In summary, these data reveal that ectonucleotidases mitigate the release of NETs by neutrophils, both at baseline and in the context of lupus.

## Discussion

The literature already hints at an intersection between adenosine signaling and lupus. One study has suggested that lupus-prone Fas^lpr/lpr^ mice are protected from nephritis by an adenosine-receptor agonist (35). In some lupus patients, there is increased adenosine deaminase activity (and presumably lower adenosine content) in blood (36, 37). Perhaps to compensate for this, adenosine-receptor density may be increased on lupus lymphocytes (38). Moreover, these pathways are potentially amenable to pharmacologic manipulation in patients. For example, adenosine signaling is indirectly amplified by a number of medications with relevance to rheumatology including methotrexate, dipyridamole, and phosphodiesterase 4 inhibitors.

Here, we have demonstrated for the first time a role for the ectonucleotidases CD39 and CD73 in protecting against lupus. Deletion of CD39 and CD73 leads to higher levels of anti-RNP antibodies in response to pristane, with CD73 deletion in particular promoting expansion of splenic B cell and T cell populations that likely contribute to autoantibody production. Within the T cell compartment, it is notable that some of these changes (expansion of effector/memory T cells and T_H_17 cells) were present independent of pristane administration, which would fit with a general predisposition toward inflammation and autoimmunity conferred by loss of ectonucleotidase activity (11, 39, 40). For example, protective roles for ectonucleotidases have been suggested in rheumatoid arthritis (41, 42), juvenile idiopathic arthritis (43), inflammatory bowel disease (44, 45), autoimmune hepatitis (46), and atherosclerosis (47, 48). This is the first study to explore these pathways in lupus.

The classic markers of Tregs are CD25 and the forkhead transcription factor FoxP3. Recently, it has also been recognized that both CD39 and CD73 are surface markers of Tregs, generating adenosine to induce anergy in effector T cells *via* the A_2A_ receptor (15, 49, 50). In contrast to the protective role of Tregs, effector T_H_17 cells have a well-established role in promoting autoimmunity in various diseases including multiple sclerosis, rheumatoid arthritis, and lupus. Indeed, lupus mice and patients have increased frequency of circulating T_H_17 cells, which correlate with disease activity (51–53). Furthermore, the induction of lupus by pristane is significantly mitigated by deletion of the IL-17 gene (54). Our data now demonstrate that deficiency of either CD39 or CD73 leads to polarization toward T_H_17 cells, which we hypothesize to be at least partially attributable to a defect in immunosuppressive Treg function in ectonucleotidase-deficient mice (15).

While this study focused on ectonucleotidases, follow-up studies should further characterize the purine species that they regulate and downstream signaling events. While the conversion of ATP to AMP can be countered by extracellular kinase activity, the conversion of AMP to adenosine can only be reversed upon intracellular transport of adenosine. This places CD73 at a crucial checkpoint in the conversion of extracellular, proinflammatory ATP into anti-inflammatory adenosine. Indeed, in animal models, CD73 has been shown to protect against LPS-induced neutrophil trafficking into lungs (55), and permeability of hypoxic endothelium to neutrophils (12–14). In our hands, CD73 deficiency, when compared with CD39 deficiency, was the greater potentiator of both autoimmune-exacerbating and inflammatory neutrophil phenotypes in the pristane model, thereby hinting that adenosine (and its downstream signaling pathways) in particular warrants further study.

It should be emphasized that our work is performed in a mouse model of lupus and has potential limitations when extrapolated to human lupus. For example, a series of families identified with CD73 null mutations did not have a clinical autoimmune phenotype, but were instead predisposed to severe calcification of lower extremity arteries and joint capsules (56). To the best of our knowledge, CD73 mutations have never been described in lupus patients. There is growing interest in the role of purinergic signaling in immune function, and as a therapeutic target. Recent work has described CD38/CD203a as an alternative mechanism of ATP catabolism by both human T cells (perhaps especially Tregs) and cancer cells (57, 58). Indeed, antagonism of CD38 is currently being explored as a therapeutic approach to boost the anticancer immune response (59, 60). Interestingly, in mice, CD38 deficiency has in some cases been shown to attenuate autoimmune/inflammatory disease (61), again emphasizing differences between these pathways in mouse and human. Now that our study has highlighted the potential importance of purinergic signaling in lupus, future studies should consider parallel/compensatory pathways such as CD38/CD203a, as well as adenosine deaminase (62, 63), in both mice and humans.

Adenosine receptors vary in both affinities for adenosine and tissue distribution (10). For example, neutrophils express all four adenosine receptors (64). The A_1_ receptor (which has a high affinity for adenosine) promotes neutrophil chemotaxis, while the other three receptors (which may only become activated when the environment is flooded with excess adenosine) tend to silence neutrophils (10, 64). In particular, the A_2A_ and A_3_ receptors are expressed at high levels on neutrophils and are recognized as suppressors of neutrophil effector functions (64–66). One study demonstrated that agonism of the A_2A_ receptor is protective against nephritis in a different model of lupus (35). However, the impact of adenosine signaling on lupus vascular disease and neutrophil activity, and the role of adenosine receptors beyond the A_2A_ receptor, are heretofore unexplored. Our data now for the first time link ectonucleotidases to the release of NETs by neutrophils. At baseline (i.e., the saline condition) both CD39^−/−^ and CD73^−/−^ mice demonstrated exaggerated NET release, which was further potentiated by pristane administration. We speculate that ectonucleotidases play a role in generating a local “halo” of adenosine that suppresses NET release.

In the 1950s, mortality attributable to lupus was more than 50%, with that startling number driven especially by renal failure. With advances in immunosuppressive therapy and transplant medicine, nephritis is now a rare cause of death. In its place, cardiovascular disease has emerged as a leading cause of mortality in lupus (27). While NETs were originally described as key players in host defense, recent work has pointed to a multifaceted (and generally deleterious) intersection with the vasculature. Proteases in NETs kill endothelial cells. NETs stimulate interferon production, which reduces the numbers and function of restorative endothelial progenitors (21, 67). Furthermore, in lupus-relevant mouse models, inhibition of NETosis mitigates both arterial and venous thrombosis (21, 34, 68). Here, we posit that neutrophil hyperactivity (in the absence of nucleotide dissipation) is an important mediator of the pristane-dependent endothelial dysfunction observed in ectonucleotidase-deficient mice, although confirmation of this will require further study.

In summary, we have revealed a previously unrecognized role for ectonucleotidases in protection against lupus. In particular, these data are the first to link ectonucleotidases with lupus autoimmunity and vascular disease. Future therapeutic strategies may harness purinergic signaling to limit the damage inflicted by lupus upon organs and blood vessels.

## Ethics Statement

This study was carried out in accordance with the recommendations of the National Research Council, Guide for the Care and Use of Laboratory Animals. The protocol was approved by the Institutional Animal Care and Use Committee.

## Author Contributions

LM, SY, GS, RA, JH, and YK conducted experiments and analyzed data. JK, YK, and DP designed the study. All the authors participated in writing the manuscript and gave approval before submission.

## Conflict of Interest Statement

The authors declare that the research was conducted in the absence of any commercial or financial relationships that could be construed as a potential conflict of interest.
