# Supplementary material for: Ectonucleotidase-Mediated Suppression of Lupus Autoimmunity and Vascular Dysfunction
Source: Front Immunol. 2018 Jun 11;9:1322. doi: 10.3389/fimmu.2018.01322 (PMC6004379; doi:10.3389/fimmu.2018.01322)
Supplement: Supplementary file 1 [file Presentation_1.PDF]

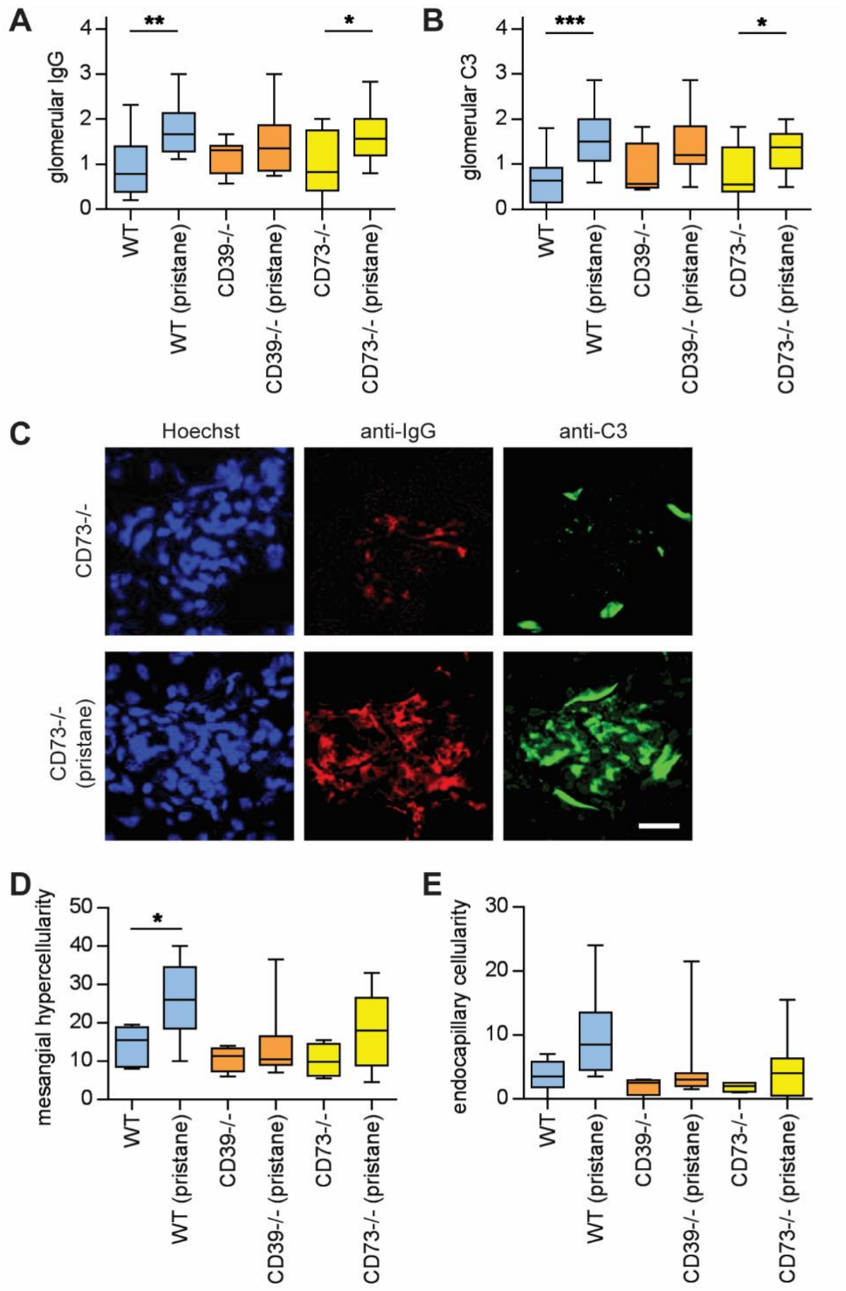


**Supplementary Figure 1** Ectonucleotidase deficiency does not modulate the modest kidney phenotype of pristane-treated C57BL/6 mice. **A,** Kidney glomeruli were scored for IgG deposition. **B,** Kidney glomeruli were scored for C3 deposition. **C,** Representative glomerular staining for panels E and F; scale bar=20 microns. **D,** Glomerular mesangial hypercellularity (as described in Methods). **E,** Glomerular endocapillary cellularity (as described in Methods). For all panels, n=5 per control group and 8-10 per pristane group. *p<0.05, **p<0.01, and ***p<0.001.

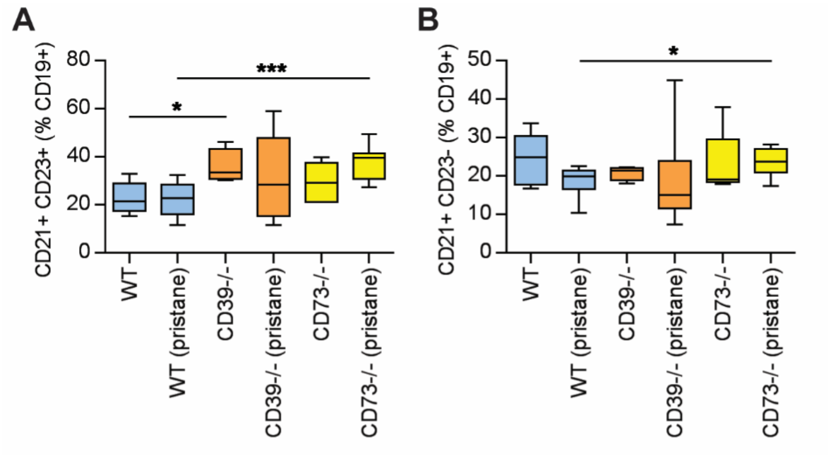


**Supplementary Figure 2** Modulation of follicular and marginal zone B cells by ectonucleotidase deficiency in pristane-treated mice. Mice were administered either saline or pristane, as indicated. 36 weeks later, splenocytes were analyzed by flow cytometry. **A,** $CD21^{+}$ $CD23^{+}$ follicular B cells, presented as the percentage of $CD19^{+}$ B cells. **B,** $CD21^{+}$ $CD23^{-}$ marginal-zone B cells, presented as the percentage of $CD19^{+}$ B cells. N=5 per control group and 8-10 per pristane group; *p<0.05 and ***p<0.001.

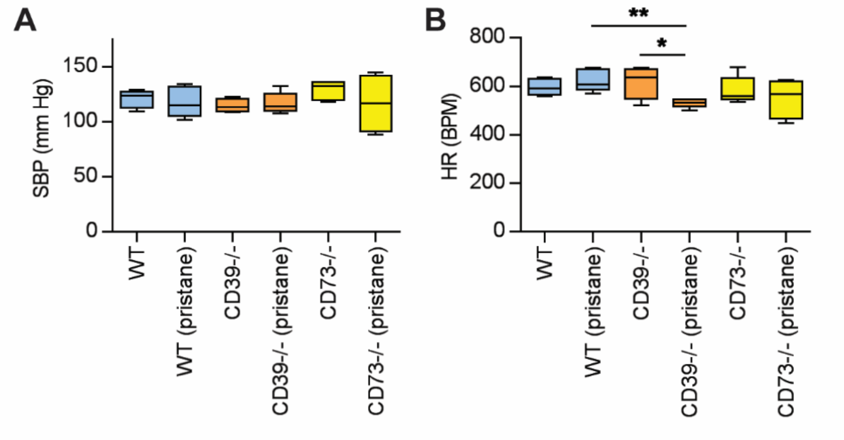


**Supplementary Figure 3** Blood pressure and heart rate are not significantly regulated by ectonucleotidase deficiency in pristane-treated mice. Mice were administered either saline or pristane, as indicated. 36 weeks later, splenocytes were analyzed by flow cytometry. **A,** Systolic blood pressure (millimeters mercury). **B,** Heart rate (beats per minute). N=5 per group; *p<0.05 and **p<0.01.

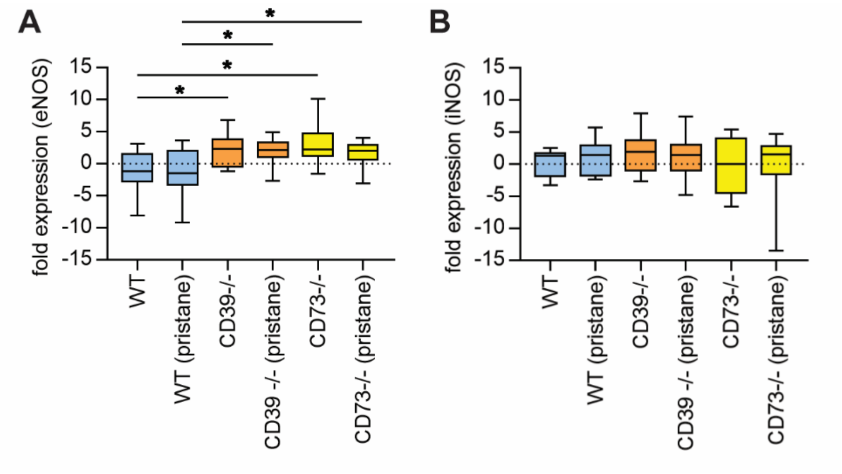


**Supplementary Figure 4** Gene expression (aorta) of endothelial nitric oxide synthase (eNOS) and inducible NOS (iNOS), as determined by quantitative PCR. Mice were administered either saline or pristane, as indicated. 36 weeks later, aortas were harvested. **A,** eNOS gene expression. **B,** iNOS gene expression. For both panels, n=7-9 control mice and 13-19 pristane mice per graph; *$p<0.05$. In panel B, no relevant comparisons were statistically significant.

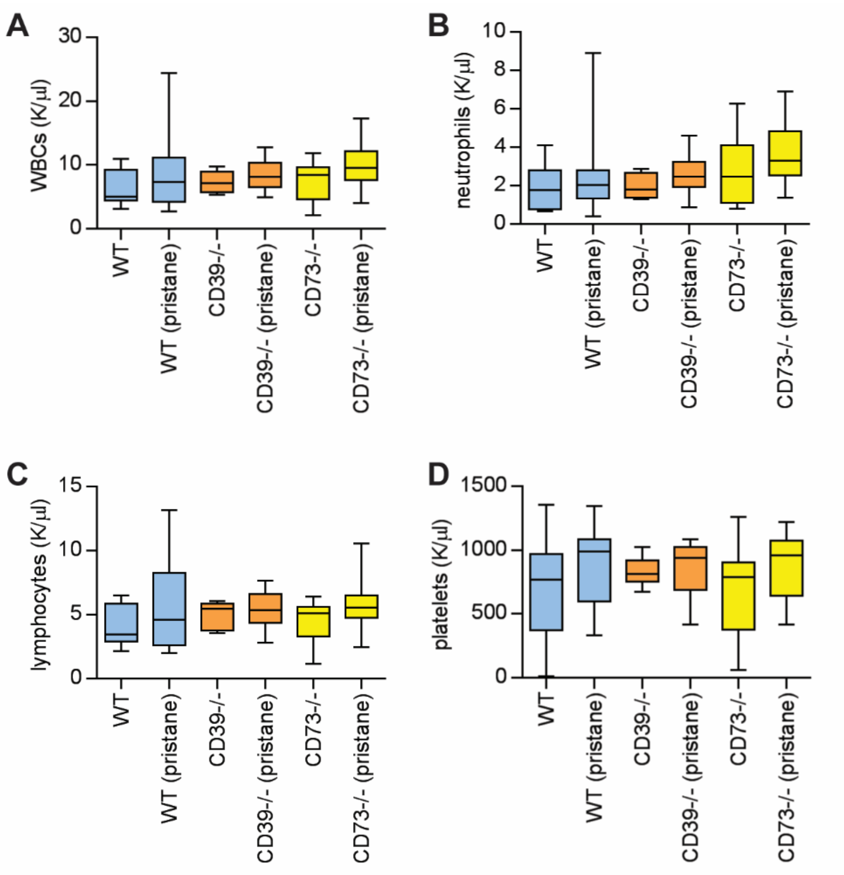


**Supplementary Figure 5** Complete blood count data for all groups of mice. WBC=total white blood cells. K=$10^3$. N=10 per control group and 17-19 per pristane group. No comparisons were statistically significant.
